# Twitter in der Kardiologie: Praktische Anleitung für den professionellen Gebrauch

**DOI:** 10.1007/s00399-020-00699-3

**Published:** 2020-07-15

**Authors:** Dominik Linz, David Duncker

**Affiliations:** 1 Maastricht Heart+Vascular Center, Maastricht University Medical Center (MUMC+), 6202 AZ Maastricht, Niederlande; 2 Rhythmologie und Elektrophysiologie, Klinik für Kardiologie und Angiologie, Medizinische Hochschule Hannover, 30625 Hannover, Deutschland

**Keywords:** Soziale Medien, Kommunikation, Networking, Kardiologische Community, Weiterbildung, Social Media, Communication, Networking, Cardiology community, Continuing education

## Abstract

Besonders Twitter hat in der kardiologischen Community an Popularität und Einfluss gewonnen. Twitter ist ein sinnvolles und dynamisches Medium zur Kommunikation, Vernetzung und Weiterbildung für forschende, lehrende und klinisch tätige Kardiologinnen und Kardiologen. Dieser Artikel soll am Beispiel von Twitter Kardiologen eine praktische Handlungsanleitung bieten, um dieses soziale Netzwerk in Zukunft erfolgreich und professionell nutzen zu können, und verdeutlichen, wie man sich über aktuelle Technologien und neue Studienergebnisse auf dem Laufenden halten kann.

## Abstract (en)

Social media is becoming increasingly popular in the cardiology community. Particularly Twitter is an emerging and dynamic medium to communicate, connect and educate academic and clinical cardiologists. This article aims to provide a practical guide how to professionally use this social network to keep yourself up-to-date about new techniques, the latest study results and news presented at national or international conferences.

Soziale Medien haben in allen gesellschaftlichen Bereichen eine immer wichtiger werdende Position eingenommen. Sie zeichnen sich durch eine hohe Geschwindigkeit eher kleiner Informationseinheiten aus. Besonders Twitter hat in der kardiologischen Community an Popularität und Einfluss gewonnen. Dieser Beitrag soll Kardiologen eine praktische Handlungsanleitung bieten, um das soziale Netzwerk Twitter als Kommunikationsplattform professionell nutzen zu können. Darüber hinaus werden nützliche Tipps gegeben werden, wie man sich über aktuelle Technologien und neue Studienergebnisse auf dem Laufenden halten kann.

Kardiologinnen und Kardiologen haben sich traditionell bislang fast ausschließlich über Fachzeitschriften oder Besuche nationaler und internationaler Kongresse oder Weiterbildungsveranstaltungen fortgebildet. In den letzten Jahren haben Online-Veranstaltungen, sog. Webinars, an Bedeutung gewonnen. Nun haben sich auch, aktuell zusätzlich beschleunigt durch die Maßnahmen zur Einschränkung sozialer Kontakte während der COVID-19-Pandemie, verschiedene soziale Medien (SoMe) wie Twitter, LinkedIn, Facebook oder WhatsApp zu neuen edukativen Medien entwickelt [1]. Lernen über SoMe ist *asynchron*, das heißt, sie können zu jeder Zeit, an jedem Ort und angepasst an die eigene Geschwindigkeit genutzt werden [2, 3]. Außerdem ist Lernen über SoMe *demokratisch*. Das bedeutet, jeder kann und darf mitreden [2, 3], jeder kann Fragen stellen und Erfahrungen austauschen und dadurch Teil einer Unterhaltung werden, wodurch es sich von dem passiven Konsumieren von Informationen in traditionellen Lernsituationen deutlich unterscheidet. Zusätzlich bietet Twitter eine optimale Plattform, um sich mit Kollegen zu vernetzen und in einen engeren und schnelleren Austausch zu treten.

Insbesondere Twitter entwickelt sich seit einigen Jahren zu einer schnellen, prägnanten und einfachen Plattform, über die fachliche und wissenschaftliche Inhalte geteilt werden [4]. Aktuell wird sogar diskutiert, ob Fortbildungsinhalte auf Twitter für ärztliche Fortbildungspunkte (Continuing Medical Education, CME) anerkannt werden sollten [5, 6]. Während der letzten 5 Jahre ist die Anzahl von Twitter Journal Clubs um 118,93 % angestiegen [7].

Neben Weiterbildung kann eine SoMe-Kampagne auf Twitter die Sichtbarkeit eines Manuskripts positiv beeinflussen [8]. In der „ESC Journals Study“ wurden 696 Publikationen randomisiert in Promotion auf Twitter oder den Kontrollarm. Manuskripte, die auf Twitter beworben wurden, wurden 1,43-fach häufiger zitiert [8]. Während Zitationen beeinflusst werden, konnte in einer anderen Studie für das Aufrufen des Artikels auf der Internetseite innerhalb von 30 Tagen durch SoMe-Aktivitäten jedoch kein Einfluss festgestellt werden [9].

Zum einen können über SoMe neue Publikationen verbreitet werden, zum anderen ist das Erwähnen von Publikationen in SoMe auch für die Zeitschriften interessant. Zeitschriften werden nicht mehr nur über den Impact Factor beurteilt, sondern auch über den Altmetric Score, der neben der Anzahl der Zitationen auch maßgeblich durch die Anzahl von Tweets und Retweets auf Twitter beeinflusst wird [10].

Zusätzlich erfolgt über SoMe mittlerweile auch eine breite Diskussion von neuen Daten und Publikationen, was als „public peer review“ bezeichnet wird und einen entscheidenden Einfluss auf Erfolg oder Misserfolg einer neuen Technik haben kann. So wurde unter den Hashtags #radialfirst oder #ldtra („left distal transradial“) ein Umdenken in der interventionellen Kardiologie in Bezug auf Standardzugangswege für Koronarangiographien angestoßen und hierbei gleichzeitig die Sicherheit und Machbarkeit des Zugangs sowie relevante wissenschaftliche Publikationen zu diesem Thema verbreitet. Ein besonders eindrucksvolles Beispiel, wie SoMe klinische Praxis und Entwicklung von neuen Therapieansätzen beeinflussen kann, stellt die Entwicklung der Technik der His-Bündel-Btimulation dar [11]. Obwohl diese Methode bereits vor mehreren Jahren beschrieben wurde, konnte sie sich nicht als Standardschrittmachertherapie durchsetzen und geriet ein wenig in Vergessenheit. Der Erfolg der kardialen Resynchronisationstherapie mittels biventrikulärer Stimulation trug hier ebenso dazu bei. Unter dem Hashtag #dontdisthehis sorgten jedoch einige Elektrophysiologen für ein exponentielles Wachstum an Tweets und Twitter-Impressionen zu diesem Thema und trugen so dazu bei, die Methode der His-Bündel-Stimulation wieder in die kardiologische Praxis zu tragen. Die #dontdisthehis-Bewegung breitete sich rasch auf Kongressen und Fortbildungsveranstaltungen aus und wurde anhand von weiteren randomisierten Studien stärker untermauert. Dieses Beispiel zeigt, welch starken Einfluss SoMe auf den klinischen Alltag haben könnte [11].

SoMe stellen eine interaktive und edukative Modalität dar, jedoch bleibt häufig unklar, wie sie am besten genutzt werden können.

## How-to: Wie nutze ich Twitter effektiv und professionell?

Twitter kann sowohl über einen Computer (Website: https://twitter.com/home) als auch über ein Smartphone (Gratis Download der Twitter App zum Beispiel über „App-Store“ oder „Google Play Store“) genutzt werden.

### Wie entwickle ich mein eigenes Profil und meine eigene Marke?

Mache Werbung für deine eigene Forschung und klinische Expertise. Jeder kann bei Twitter ein individuelles Profil erstellen. Das Profil auf Twitter wird „Handle“ genannt und wird in einem Tweet in Verbindung mit einem @ Zeichen genutzt (z. B. @Dominik_Linz oder @DavidDuncker). In den SoMe wird der eigene Charakter geformt durch das, was man schreibt. Daher sollte man professionell, respektvoll und freundlich agieren. Zudem sollte man seine eigene Identität verwenden. Es ist nicht nur wichtig, was man sagt, sondern auch wer man ist.

### Wie baue ich ein Tweet auf?

**Figure 1:**
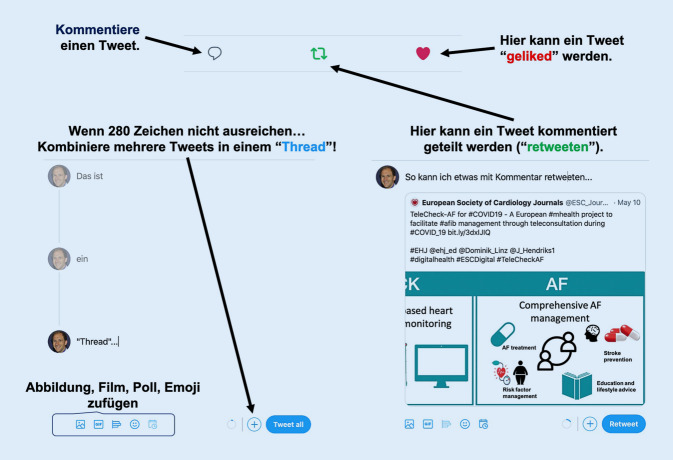

Die wichtigsten Funktionalitäten von Twitter werden in Abb. 1 zusammengefasst.

Ein Tweet ist eine sehr kurze und prägnante Zusammenfassung. Häufig ist es eine Herausforderung, in 280 Zeichen einen komplexen Gedankengang auszudrücken. Jedoch erhalten interessanterweise kürzere Beiträge auf Twitter (71 und 100 Zeichen) die meisten Retweets. Es können mehrere Tweets (max. 25) eines Nutzers zu *Threads* verkettet werden. Ein Thread kann die Basis für ein sog. Tweetorial sein. Tweetorials werden häufig genutzt, um praktische Grundlagen von Punktionstechniken oder eine Anleitung zur statistischen Auswertung von Daten zu präsentieren. Über Threads und Tweetorials soll Nutzern die Möglichkeit gegeben werden, längere Beiträge zu veröffentlichen.

#### Follower.

Will man zukünftig über Beiträge anderer Nutzer informiert werden, kann man ihnen *folgen*. Ein Nutzer, der einem anderen folgt, wird als *Follower* bezeichnet. So abonniert man den Nutzer entsprechend, und dessen Tweets werden daraufhin in der eigenen Timeline angezeigt.

#### Hashtags.

Ein Hashtag (z. B. #radialfirst) hebt ein Wort oder eine Zeichenkette in einem Tweet hervor. Hashtags werden im Allgemeinen direkt in die eigentliche Nachricht eingefügt. Über die Benutzung eines Hashtags kann ein Archiv von Tweets generiert werden. Über „Follow #radialfirst“ kann man andere Menschen auf einen Hashtag aufmerksam machen.

#### Infobox 1

Häufig verwendete Hashtags in der Kardiologie sind zum Beispiel:

*#CardioTwitter, #EPeeps, #EchoFirst, #radialfirst, #dontdisthehis, *etc.

#### Retweeten.

Wer den Tweet eines anderen mit seinen Followern teilen möchte, kann ihn *retweeten*. Dafür bietet Twitter eine integrierte Retweetfunktion an. Außerdem kann man auch Tweets zitieren. Dadurch ist ein Retweet mit Kommentar möglich.

#### Likes.

Nutzer haben die Möglichkeit, einen Tweet zu *liken*. Für das *Liken* haben Nutzer unterschiedliche Motivationen, dennoch bringen sie dadurch meist zum Ausdruck, dass ihnen etwas gefällt, sie etwas zustimmen oder sie etwas unterstützen.

Tweets werden in erster Linie den Followern eines Benutzers angezeigt, vor allem über Hashtags oder Verlinkungen/Retweets kann aber auch ein breiteres Publikum erreicht werden. Daher sollte man einem guten Tweet Hashtags (mit #), Links (als URL), Verweise auf andere Nutzerprofile „Handle“ (mit @) sowie Bilder (als URL oder direkt eingefügt) hinzufügen.

### Wie zitiere ich ein Manuskript oder füge meinem Tweet ein Video zu?

Kommentiert man einen wissenschaftlichen Artikel oder will man seine eigene aktuelle Publikation promoten, sollte man einen Link zu der Arbeit in dem Tweet erwähnen. Da Internetadressen zu einem Artikel häufig sehr lang sind, kann man einen „URL-shortener“ verwenden, um die nötigen Zeichen zu vermindern. Zusätzlich sollte man versuchen den Twitter-Handle der Erst- und/oder Letztautoren ausfindig zu machen. Dasselbe gilt für den Journal Handle.

Auch Videos können in einen Tweet integriert werden. Diese können im Format MP4 oder MOV mit einer maximalen Länge von 2,20 min hochgeladen werden. Muss ein Video noch in das MB4-Format konvertiert werden, kann ein „Online Converter“ verwendet werden.

#### Infobox 2

Ein Beispiel für ein „URL-shortener“ ist Bitly: https://bitly.com

Ein Beispiel für ein „Online Converter“ ist: https://convert-video-online.com/de/

### Wie stimuliere ich zusätzliche Interaktion?

In sozialen Medien geht es um Vernetzung und Kommunikation. Um den Austausch anzuregen, kann man die Kolleginnen und Kollegen, die man besonders ansprechen möchte, im Tweet mit ihrem jeweiligen Twitter-Handle erwähnen oder bis zu 10 Twitter-Handle mit einer Abbildung oder einem Foto verknüpfen. Eine gezielte Interaktion kann man auch durch Umfragen (Poll) in Tweets erreichen. Durch eine einfache Auswahl an bis zu vier möglichen Antworten auf eine gezielte Frage ist die Schwelle, dass Leser des Tweets mit diesem interagieren, niedrig. Nach Abschluss der Umfrage sollte das Ergebnis nach Möglichkeit zusammengefasst und kommentiert werden, um einen Mehrwehrt und eine Message zu generieren.

### Wann tweete ich am besten?

Twitter ist ein sehr schnelllebiges Medium. Die Lebensspanne eines Tweets beträgt nur etwa 18 min. Daher ist im Fall von Twitter ein wiederholtes Publizieren (retweeten) sicherlich sinnvoll (z. B. um 9:00, 12:00, 15:00, 18:00 Uhr). Der optimale Zeitpunkt eines Tweets hängt vom Publikum ab. Zeitunterschiede zu internationalen Followern sollten berücksichtigt werden.

Wenn man über einen spannenden klinischen Fall tweeten will, sollte man auf einige Punkte achten. Twitter ist ein öffentliches Medium. Man sollte niemals einen klinischen Fall am selben Tag tweeten. Auch Patienten und ihre Familie können unter den eigenen Followern sein. Unbedingt sollte außerdem darauf geachtet werden, dass keine identifizierbaren Daten getweetet werden. Öffentliche Diskussionen mit Patienten auf Twitter sollten vermieden werden.

### Wie halte ich mich über Twitter auf dem Laufenden?

#### Den großen Fachzeitschriften folgen.

Alle relevanten Journale haben eine Präsenz auf Twitter. Beispiele für Journal Handles sind in Tab. 1 dargestellt. Einige der großen kardiologischen Zeitschriften wie *European Heart Journal oder Circulation* bieten freien Zugang zu ausgewählten Artikeln, wenn der Zugriff über SoMe erfolgt.

**Table 1:** 

Journal Name	Journal Handle
*New England Journal of Medicine*	@NEJM
*Journal of the American College of Cardiology*	@JACCJournals
*ESC Journale (European Heart Journal, EUROPACE etc.)*	@ESC_Journals
*Journal of the American Medical Association & JAMA Cardiology*	@JAMA_current, @JAMACardio
*The Lancet*	@TheLancet
*Circulation*	@CircAHA

#### Den wissenschaftlichen Fachgesellschaften folgen.

European Society of Cardiology (@escardio), Deutsche Gesellschaft für Kardiologie (@DGK_org), The American College of Cardiology (@ACCinTouch), The American Heart Association (@AHAScience).

#### Sog. SoMe-Influencern folgen.

„Key Opinion Leader“ in der Kardiologie werden in Tab. 2 zusammengefasst.

**Table 2:** 

International	Deutschland
Eric Topol (@EricTopol, 256.300 Follower)	Stephan Achenbach (@Steph_Achenbach, 5142 Follower)
Mamas Mamas (@mmamas1973, 25.800 Follower)	Thomas Münzel (@tmuenzel, 1397 Follower)
David Albert (@DrDave01, 15.200 Follower)	Martin Halle (@ProfessorHalle, 1397 Follower)
John Mandrola (@drjohnm, 40.500 Follower)	Thomas Deneke (@EPDeneke, 1136 Follower)
Edward Schloss (@EJSMD, 12.500 Follower)	Philipp Sommer (@Phiso_de, 1064 Follower)
C Michael Gibson (@CMichaelGibson, 445.600 Follower)	Holger Nef (@HolgerNef, 795 Follower)
Pascal Meier (@DrPascalMeier, 113.200 Follower)	Erik Rafflenbeul (@KardiologieHH, 730 Follower)

#### Twitter während Kongressen folgen.

Auf den großen kardiologischen Kongressen findet sich eine erhebliche und zunehmende Twitteraktivität. Hierbei zeigen die Daten vom ESC Kongress 2018, dass 81 % der Tweets kongressbezogene Fortbildungsinhalte transportieren, nur 5 % zeigen tatsächlich *soziale* Aspekte [12, 13]. Über den Gebrauch von Hashtags (#ACC20, #ESCCongress, #DGKOnline2020) wird es immer einfacher, virtuell an Konferenzen teilzunehmen [13]. Dies ist insbesondere interessant für diejenigen, die aufgrund von Zeit- oder Ressourcenlimitationen nicht an den Veranstaltungen teilnehmen können.

**Figure 2:**
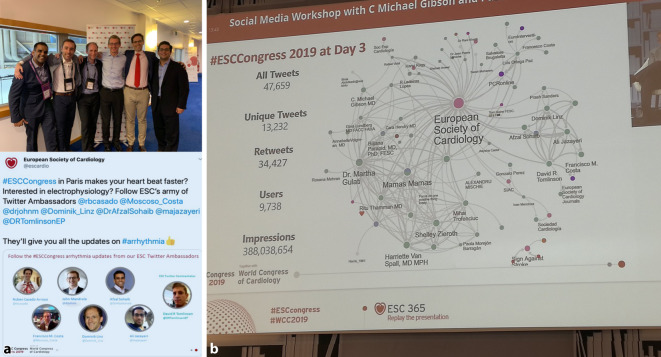

Eine neu initiierte Aktivität ist der „Twitter-Ambassador“. SoMe-Influencer werden von Kongressen und Fachgesellschaften eingeladen, aktiv über Neuigkeiten und aktuelle Vorträge zu tweeten (Abb. 2a). Während einzelne Sitzungsräume auf den Kongressen manchmal nur weniger als 100 Personen zuließen, werden Abbildungen, Präsentationsausschnitte oder einzelne Aussagen innerhalb weniger Minuten in der ganzen Welt verbreitet. Das Ausmaß und die Reichweite von Twitter-Aktivitäten können mit Hilfe einer analytischen SoMe-Plattform (z. B. www.symplur.com) evaluiert werden. In Abb. 2b wird das Ergebnis eines analytischen Mappings der Tweeter-Aktivität und der resultierenden Vernetzungen während des ESC-Kongresses 2019 in Paris gezeigt. Auch die DGK hat seit 2020 Twitter-Ambassadoren (#DGKAmbassadors) berufen.

**Figure 3:**
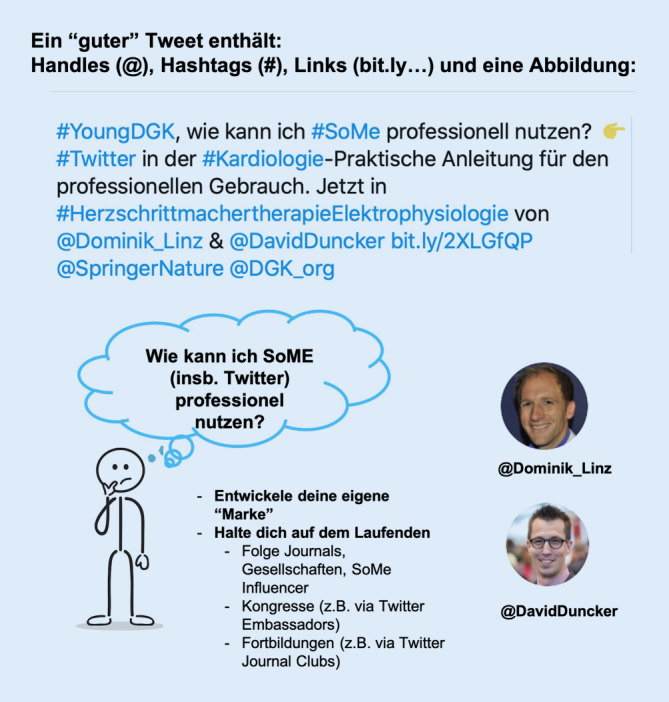

### Wie kann ich meine Tweets archivieren?

Twitter kann helfen, Likes oder Tweets oder Retweets zu archivieren. Das ist insbesondere praktisch während einer Konferenz. Alle Tweets können in einem PDF-Format exportiert werden. Über die Suchfunktion können dann Stichworte oder Hashtags gefunden werden.

#### Infobox 3

Tweets können über allmytweets.net archiviert werden, und Stichworte oder Hashtags können über die Suchfunktion gefunden werden.

## Limitationen von Twitter

Trotz der immer wichtiger werdenden Rolle von SoMe und insbesondere Twitter in der Kardiologie, ergeben sich auch einige Limitationen und Gefahren bei der professionellen Nutzung von SoMe. Twitter ist ein *offenes System*, das heißt, nicht nur Kollegen können Beiträge lesen, sondern auch Patienten, Angehörige, Politiker oder Anwälte. Eine Leitlinie zur Nutzung von SoMe in Krankhäusern wurde kürzlich von der American Hospital Association publiziert [14].

Komplexe Sachverhalte (wie häufig in der Medizin) sind nicht immer gut auf 280 Zeichen darzulegen. Dies resultiert oft in Informationsverlust oder reduzierter Differenziertheit. Anders als bei wissenschaftlichen Veröffentlichungen oder Vorträgen müssen keine Interessenskonflikte dargelegt werden. Gerade bei professionellen Inhalten oder in der Lehre ist in anderen Zusammenhängen die Kenntnis der Zielgruppe wichtig, um den Inhalt und den Ausdruck anzupassen. Anders als bei Vorträgen vor Studenten, Kollegen oder Patienten, kennt man bei Twitter aber diese Zielgruppe nicht, da die Beiträge von jedem eingesehen werden können. Im Vergleich zu anderen Bevölkerungsgruppen ist der Anteil an Twitternutzern unter Kardiologinnen und Kardiologen sowie Wissenschaftlern in Deutschland eher gering.

Wissenschaftlicher Erfolg sollte aber auch nicht ausschließlich anhand von SoMe-Aktivitäten oder Tweetzahlen gemessen werden. Gerade in SoMe kann sonst auch eine Diskrepanz zwischen tatsächlichem wissenschaftlichem Einfluss (insbesondere durch Publikationen) und der Anzahl der Twitter-Follower entstehen (sog. Kardiashian-Index; [15]). Bei Twitter-aktiven Kardiologen korreliert interessanterweise im Allgemeinen die Anzahl der Publikationen relativ gut mit der Anzahl der Twitter-Follower und „Kardiologen-Kardashians“ gibt es nur selten [16].

## Fazit für die Praxis

- Twitter ist ein sinnvolles und dynamisches Medium zur Kommunikation, Vernetzung und Weiterbildung für forschende, lehrende und klinisch tätige Kardiologinnen und Kardiologen.
- Twitter verändert die Art, wie wir uns fortbilden und wie wir uns vernetzen.
- Die Beachtung einfacher praktischer Handlungsanleitungen erleichtert erfolgreiches Tweeten (Abb. 3).

## References

[1] Clavier T, Ramen J, Dureuil B, Veber B, Hanouz JL, Dupont H, Lebuffe G, Besnier E, Compere V (2019). Use of the smartphone app WhatsApp as an E-learning method for medical residents: multicenter controlled randomized trial. JMIR Mhealth Uhealth.

[2] Alraies MC, Raza S, Ryan J (2018). Twitter as a new core competency for cardiologists. Circulation.

[3] Parwani P, Choi AD, Lopez-Mattei J, Raza S, Chen T, Narang A, Michos ED, Erwin JP, Mamas MA, Gulati M (2019). Understanding social media: opportunities for cardiovascular medicine. J Am Coll Cardiol.

[4] Sinnenberg L, DiSilvestro CL, Mancheno C, Dailey K, Tufts C, Buttenheim AM, Barg F, Ungar L, Schwartz H, Brown D, Asch DA, Merchant RM (2016). Twitter as a potential data source for cardiovascular disease research. JAMA Cardiol.

[5] Thamman R, Gulati M, Narang A, Utengen A, Mamas MA, Bhatt DL (2020). Twitter-based learning for continuing medical education?. Eur Heart J.

[6] Cabrera D, Roy D, Chisolm MS (2018). Social media scholarship and alternative metrics for academic promotion and tenure. J Am Coll Radiol.

[7] Bolderston A, Watson J, Woznitza N, Westerink A, Di Prospero L, Currie G, Beardmore C, Hewis J (2018). Twitter journal clubs and continuing professional development: an analysis of a #MedRadJClub tweet chat. Radiography (Lond).

[8] Ladeiras-Lopes R, Clarke S, Vidal-Perez R, Alexander M, Lüscher TF, ESC (European Society of Cardiology) Media Committee, European Heart Journal (2020). Twitter promotion predicts citation rates of cardiovascular articles: a preliminary analysis from the ESC Journals Randomized Study. Eur Heart J.

[9] Fox CS, Bonaca MA, Ryan JJ, Massaro JM, Barry K, Loscalzo J (2015). A randomized trial of social media from Circulation. Circulation.

[10] Barakat AF, Nimri N, Shokr M, Mahtta D, Mansoor H, Mojadidi MK, Mahmoud AN, Senussi M, Masri A, Elgendy IY (2018). Correlation of altmetric attention score with article citations in cardiovascular research. J Am Coll Cardiol.

[11] Beer D, Dandamudi G, Mandrola JM, Friedman PA, Vijayaraman P (2019). His-bundle pacing: impact of social media. Europace.

[12] Hudson S, Mackenzie G (2019). ‘Not your daughter’s Facebook’: Twitter use at the European Society of Cardiology Conference 2018. Heart.

[13] Tanoue MT, Chatterjee D, Nguyen HL, Sekimura T, West BH, Elashoff D, Suh WH, Han JK (2018). Tweeting the meeting. Circ Cardiovasc Qual Outcomes.

[14] American Hospital Association A hospital leadership guide to digital and social media engagement. https://www.aha.org/system/files/2018-04/guidetosocialmedia.pdf. Zugegriffen: 10.06.2020

[15] Hall N (2014). The Kardashian index: a measure of discrepant social media profile for scientists. Genome Biol.

[16] Khan MS, Shahadat A, Khan SU, Ahmed S, Doukky R, Michos ED, Kalra A (2020). The Kardashian index of cardiologists: celebrities or experts?. JACC Case Rep.

